# Supplementary material for: The transcription factor AP2XI-2 is a key negative regulator of Toxoplasma gondii merogony
Source: Nat Commun. 2024 Jan 26;15:793. doi: 10.1038/s41467-024-44967-z (PMC10817966; doi:10.1038/s41467-024-44967-z)
Supplement: Supplementary file 1 — Supplementary Information [file 41467_2024_44967_MOESM1_ESM.pdf]

Supplementary Information

The transcription factor AP2XI-2 is a key negative regulator of *Toxoplasma gondii* merogony

Jin-Lei Wang[1✉], Ting-Ting Li[1], Nian-Zhang Zhang[1], Meng Wang[1], Li-Xiu Sun[1], Zhi-Wei Zhang[1], Bao-Quan Fu[1], Hany M. Elsheikha[2✉] & Xing-Quan Zhu[3✉]

[1]State Key Laboratory for Animal Disease Control and Prevention, Key Laboratory of Veterinary Parasitology of Gansu Province, Lanzhou Veterinary Research Institute, Chinese Academy of Agricultural Sciences, Lanzhou, Gansu Province 730046, People's Republic of China

[2]Faculty of Medicine and Health Sciences, School of Veterinary Medicine and Science, University of Nottingham, Sutton Bonington Campus, Loughborough, LE12 5RD, UK

[3]Laboratory of Parasitic Diseases, College of Veterinary Medicine, Shanxi Agricultural University, Taigu, Shanxi Province 030801, People's Republic of China

✉emails: xingquanzhu1@hotmail.com (X.Q.Z.); hany.elsheikha@nottingham.ac.uk (H.M.E); and wangjinlei90@126.com (J.L.W.)

Supplementary Figures

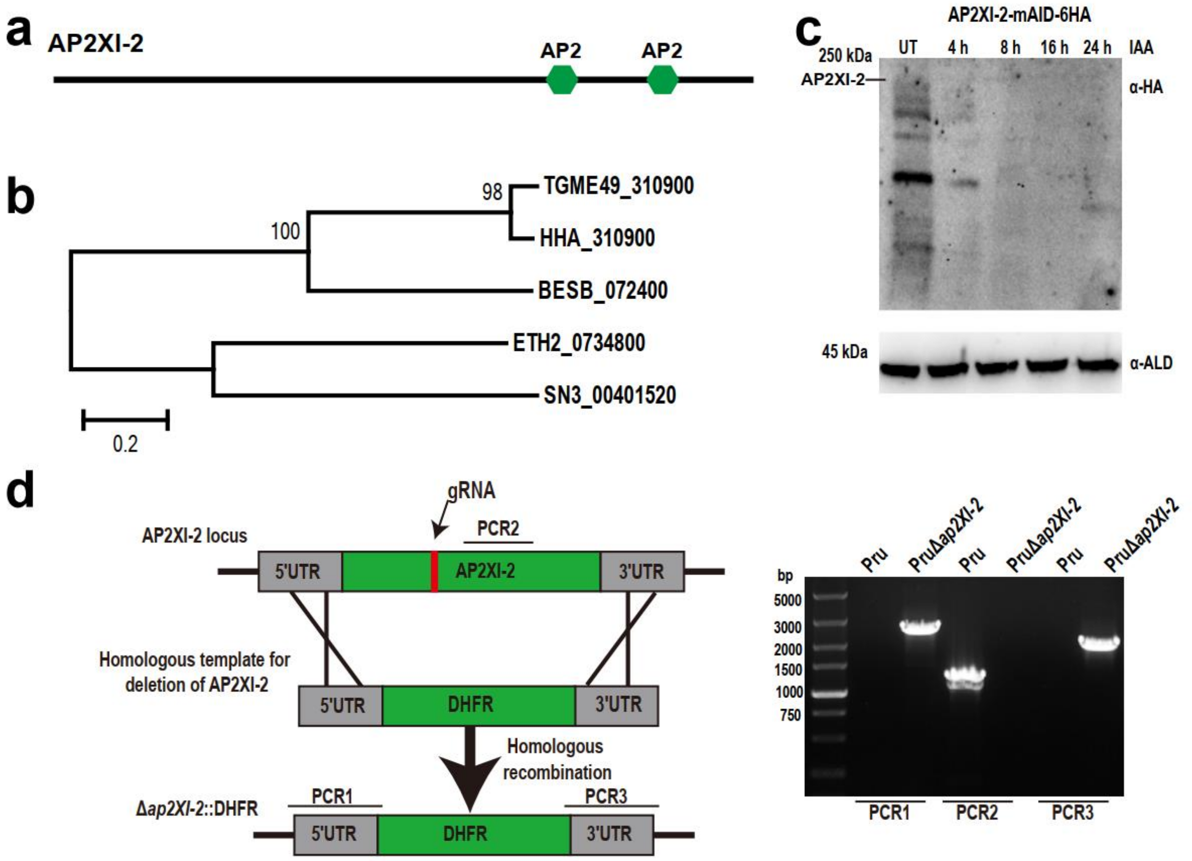

Supplementary Fig. 1 Construction and characterization of the AP2XI-2 deletion strains.

a. Illustration showing the organization of the AP2 (APETALA2) domain of AP2XI-2. **b.** Phylogenetic analysis of AP2XI-2 in Apicomplexa. *Toxoplasma gondii* (TGME49_310900), *Hammondia hammondi* (HHA_310900), *Besnoitia besnoiti* (BESB_072400), *Eimeria tenella* (ETH2_0734800), and *Sarcocystis neurona* (SN3_00401520). The phylogenetic analysis was performed using MEGA 7.0 with a maximum likelihood method and LG model of amino acid substitution. The final tree was condensed with a cut-off value 50% and the numbers displayed on the nodes represent bootstrap values from 1,000 replicates. **c.** Western blotting analysis for verifying the degradation of AP2XI-2-mAID-6HA protein after addition of auxin (500 μM IAA) for different time durations. AP2XI-2-mAID-6HA protein was detected with anti-HA. The breakdown products of the tagged protein detected by immunoblotting may reflect instability in the parasite lysate or posttranscriptional modifications. Anti-aldolase (ALD) antibodies served as a loading control. **d.** Schematic representation of knocking out AP2XI-2 by CRISPR-Cas9 mediated homologous gene replacement. PCR1 and PCR3 validated the 5′ and 3′ integration of the selection cassette, whereas PCR2 confirmed the successful deletion of the *ap2XI-2* gene. PCRs confirmed the successful disruption of *ap2XI-2* gene in PruΔ*ku80*Δ*hxgprt* strain. Source data are provided as a Source data file.

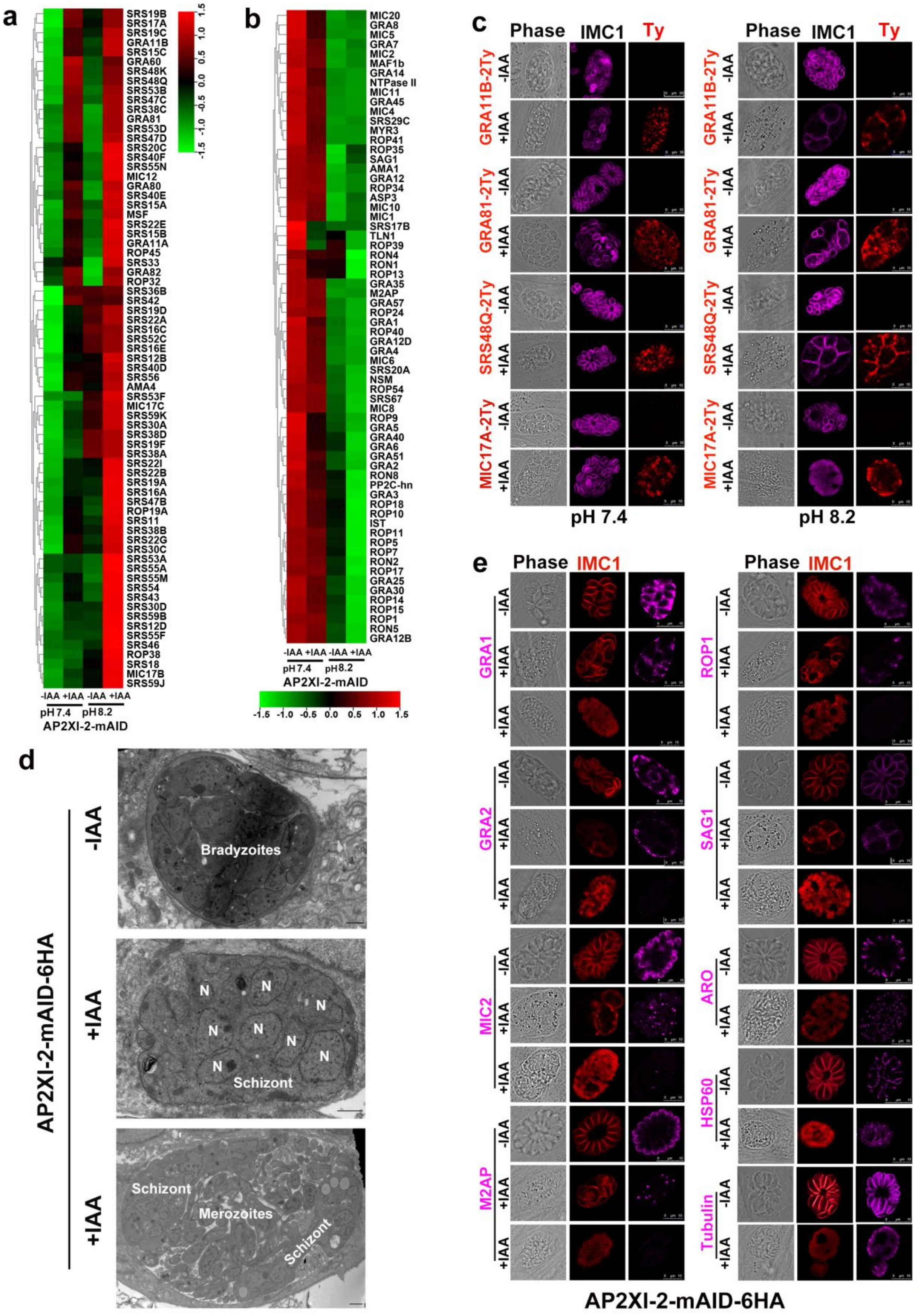
a
SRS19B
SRS17A
SRS19C
GRA11B
SRS15C
GRA60
SRS48K
SRS48Q
SRS53B
SRS47C
SRS38C
GRA81
SRS53D
SRS47D
SRS20C
SRS40F
SRS55N
MIC12
GRA80
SRS40E
SRS15A
MSF
SRS22E
SRS15B
GRA11A
ROP45
SRS33
GRA82
ROP32
SRS36B
SRS42
SRS19D
SRS22A
SRS16C
SRS52C
SRS16E
SRS12B
SRS40D
SRS56
AMA4
SRS53F
MIC17C
SRS59K
SRS30A
SRS38D
SRS19F
SRS38A
SRS22I
SRS22B
SRS19A
SRS16A
SRS47B
ROP19A
SRS11
SRS38B
SRS22G
SRS30C
SRS53A
SRS55A
SRS55M
SRS54
SRS43
SRS30D
SRS59B
SRS12D
SRS55F
SRS46
ROP38
SRS18
MIC17B
SRS59J
1.5 1.0 0.5 0.0 -0.5 -1.0 -1.5
-IAA +IAA -IAA +IAA
pH 7.4 pH 8.2
AP2XI-2-mAID
b
MIC20
GRA8
MIC5
GRA7
MIC2
MAF1b
GRA14
NTPase II
MIC11
GRA45
MIC4
SRS29C
MYR3
ROP41
ROP35
SAG1
AMA1
GRA12
ROP34
ASP3
MIC10
MIC1
SRS17B
TLN1
ROP39
RON4
RON1
ROP13
GRA35
M2AP
GRA57
ROP24
GRA1
ROP40
GRA12D
GRA4
MIC6
SRS20A
NSM
ROP54
SRS67
MIC8
ROP9
GRA5
GRA40
GRA6
GRA51
GRA2
RON8
PP2C-hn
GRA3
ROP18
ROP10
IST
ROP11
ROP5
ROP7
RON2
ROP17
GRA25
GRA30
ROP14
ROP15
ROP1
RON5
GRA12B
-IAA +IAA -IAA +IAA
pH 7.4 pH 8.2
AP2XI-2-mAID
-1.5 -1.0 -0.5 0.0 0.5 1.0 1.5
c
Phase IMC1 Ty
Phase IMC1 Ty
GRA11B-2Ty
GRA81-2Ty
SRS48Q-2Ty
MIC17A-2Ty
-IAA
+IAA
pH 7.4
pH 8.2
d
AP2XI-2-mAID-6HA
-IAA
+IAA
+IAA
Bradyzoites
N
Schizont
Schizont
Merozoites
Schizont
e
Phase IMC1
Phase IMC1
GRA1
GRA2
MIC2
M2AP
ROP1
SAG1
ARO
HSP60
Tubulin
-IAA
+IAA
AP2XI-2-mAID-6HA

Supplementary Fig. 2 Depletion of AP2XI-2 activates the expression of merozoite restricted genes and represses the expression of tachyzoite genes.

a. Heat map showing the increased expression of selected merozoite highly expressed genes after depletion of AP2XI-2. The color scale indicates $\log_2$-transformed fold changes. **b.** Heat map showing the decreased expression of genes typically highly expressed in tachyzoites after depletion of AP2XI-2. The color scale indicates $\log_2$-transformed fold changes. **c.** Depletion of AP2XI-2 activates the merozoite-specific proteins GRA11B, GRA81, SRS48Q and MIC17A as determined by IFA in the indicated parasites treated with IAA for 3 days under neutral (left) or alkaline medium (right). Magenta, anti-IMC1; red, anti-Ty. Scale bar, 10 μm. **d.** Representative transmission electron microscopic micrographs of AP2XI-2-depleted parasites under an alkaline condition for 3 days, showing AP2XI-2-depleted parasites undergoing merogony to produce mature merozoites. N, nucleus. **e.** Depletion of AP2XI-2 represses the tachyzoite proteins GRA1, GRA2, MIC2, M2AP, ROP1, and SAG1 as determined by IFA of AP2XI-2-expressing parasites under neutral medium for 32 h or AP2XI-2-depleted parasites treated with IAA for 3 days under alkaline medium. Scale bar, 10 μm.

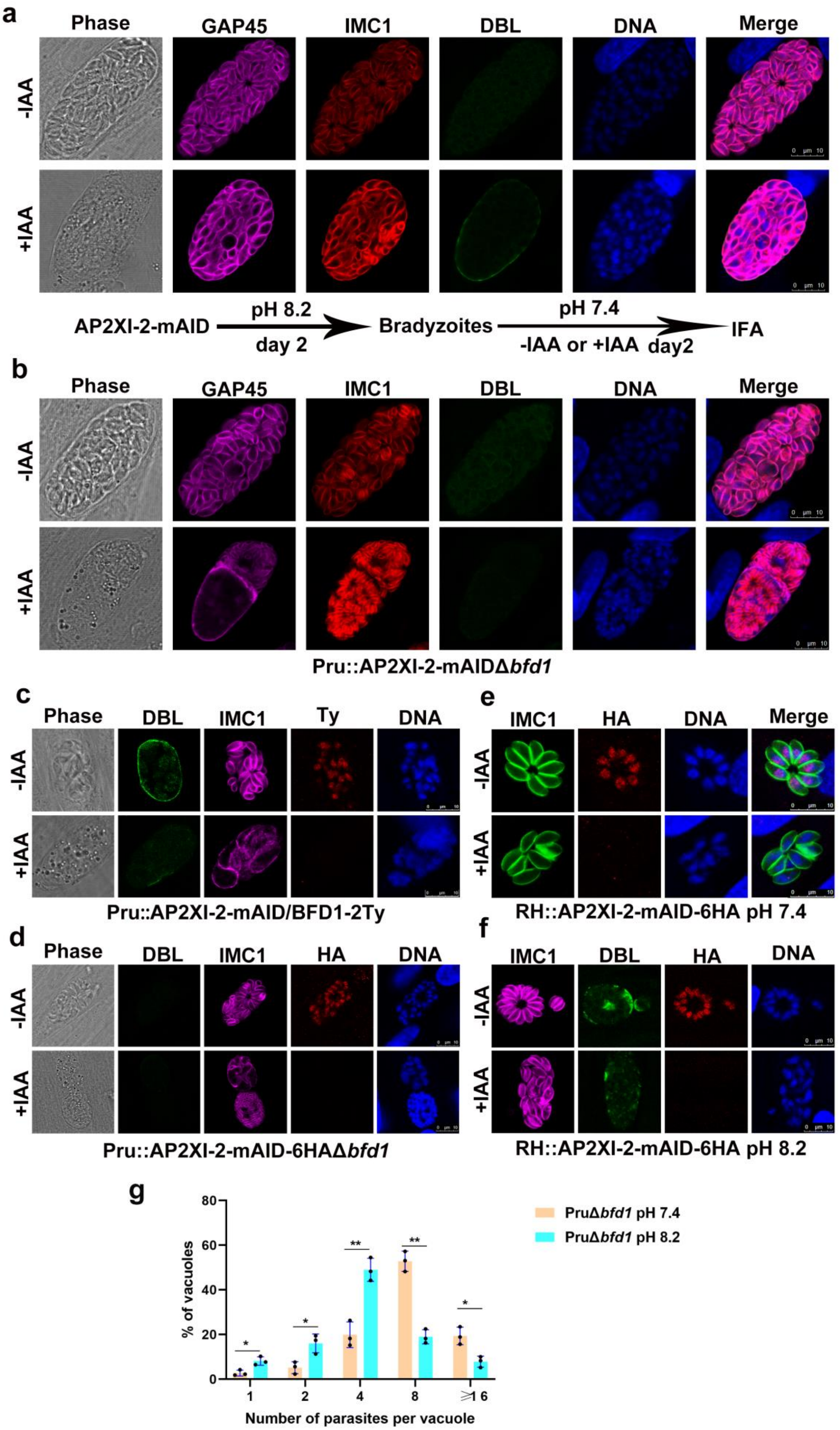
a
Phase
GAP45
IMC1
DBL
DNA
Merge
-IAA
+IAA
AP2XI-2-mAID
pH 8.2
day 2
Bradyzoites
pH 7.4
-IAA or +IAA day2
IFA
b
Pru::AP2XI-2-mAIDΔbfd1
c
Ty
Pru::AP2XI-2-mAID/BFD1-2Ty
d
HA
Pru::AP2XI-2-mAID-6HAΔbfd1
e
RH::AP2XI-2-mAID-6HA pH 7.4
f
RH::AP2XI-2-mAID-6HA pH 8.2
g
% of vacuoles
Number of parasites per vacuole
PruΔbfd1 pH 7.4
PruΔbfd1 pH 8.2

Supplementary Fig. 3 Bradyzoite stage is not a mandatory step for the AP2XI-2-depleted parasite to undergo merogony and produce mature merozoites under alkaline conditions.

a. AP2XI-2-depleted parasite did not require the transition through the bradyzoite stage to commit into merozoites. The AP2XI-2-expressing tachyzoites were allowed to infect HFFs for 4 h followed by incubation in an alkaline medium without CO_2 for 2 days for induction of bradyzoites, then the medium was changed to neutral media for 2 additional days with or without IAA. Magenta, anti-GAP45; red, anti-IMC1; green, FITC-DBL. Scale bar, 10 µm. **b.** Disruption of *bfd1* in the AP2XI-2-depleted parasites did not affect the parasites to undergo merogony and produce mature merozoites induced by exposure to alkaline stress. The Pru::AP2XI-2-mAID-6HAΔ*bfd1* parasites were allowed to infect HFFs for 4 h followed by incubation in an alkaline culture medium without CO_2 for 3 days with or without IAA. Magenta, anti-GAP45; red, anti-IMC1; green, FITC-DBL. Scale bar, 10 µm. **c.** Depletion of AP2XI-2 affects the expression of BFD1 under alkaline conditions. The indicated parasites were allowed to infect HFFs for 4 h followed by incubation in an alkaline medium without CO_2 for 3 days with or without IAA. Green, FITC-DBL; magenta, anti-IMC1; red, anti-Ty. Scale bar, 10 µm. **d.** Disruption of *bfd1* did not affect the expression of the AP2XI-2. The indicated parasites were allowed to infect HFFs for 4 h followed by incubation in an alkaline media without CO_2 for 3 days with or without IAA. Green, FITC-DBL; magenta, anti-IMC1; red, anti-HA. Scale bar, 10 µm. **e, f.** Depletion of AP2XI-2 in type I RH did not trigger tachyzoite-to-merozoite switch. The RH::AP2XI-2-mAID-6HA parasite was grown in neutral medium for 24 h (**e**) or allowed to infect HFFs for 2 h followed by incubation in an alkaline medium without CO_2 for 2 days (**f**) with or without IAA. Parasites were stained with anti-IMC1 and AP2XI-2 were detected by anti-HA. Bradyzoite cyst wall was stained with FITC-DBL. Scale bar, 10 µm. **g.** Quantification of the replication of the PruΔ*bfd1* strain was conducted in HFFs for 30 h under both neutral and alkaline conditions (the alkaline medium was added 2 h post-invasion of parasites in neutral medium). Data represents the mean ± SD from three independent experiments, analyzed by two-tailed, unpaired *t* test, $*p = 0.0263$ for 1 parasite per vacuole; $*p = 0.0327$ for 2 parasites per vacuole; $**p = 0.0073$ for 4 parasites per vacuole; $**p = 0.0018$ for 8 parasites per vacuole; $*p = 0.0227$ for ≥ 16 parasites per vacuole.

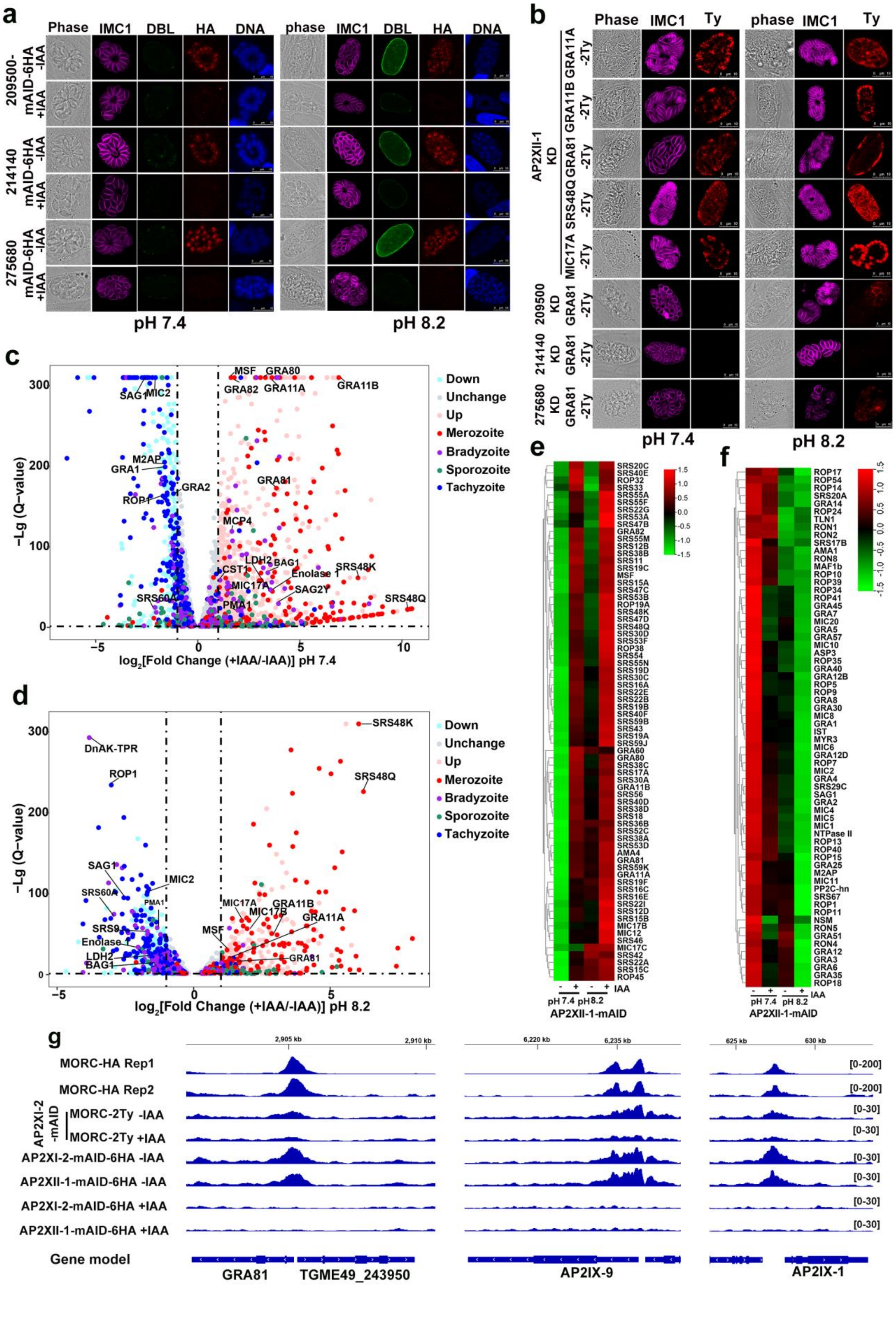
a
Phase
IMC1
DBL
HA
DNA
phase
209500-mAID-6HA -IAA
209500-mAID-6HA +IAA
214140 mAID-6HA -IAA
214140 mAID-6HA +IAA
275680 mAID-6HA -IAA
275680 mAID-6HA +IAA
pH 7.4
pH 8.2
b
Ty
AP2XII-1 KD
GRA11A -2Ty
GRA11B -2Ty
GRA81 -2Ty
SRS48Q -2Ty
MIC17A -2Ty
209500 KD GRA81 -2Ty
214140 KD GRA81 -2Ty
275680 KD GRA81 -2Ty
c
Down
Unchange
Up
Merozoite
Bradyzoite
Sporozoite
Tachyzoite
-Lg (Q-value)
log2[Fold Change (+IAA/-IAA)] pH 7.4
MSF
GRA80
GRA82
GRA11A
GRA11B
SAG1
MIC2
M2AP
GRA1
GRA2
ROP1
GRA81
MCP4
LDH2
BAG1
CST1
MIC17A
Enolase 1
SRS48K
SAG2Y
SRS60A
PMA1
SRS48Q
d
log2[Fold Change (+IAA/-IAA)] pH 8.2
SRS48K
DnAK-TPR
ROP1
SRS48Q
SAG1
MIC2
SRS60A
PMA1
SRS9
Enolase 1
LDH2
BAG1
MIC17A
MIC17B
GRA11B
GRA11A
MSF
GRA81
e
pH 7.4
pH 8.2
IAA
AP2XII-1-mAID
f
pH 7.4
pH 8.2
IAA
AP2XII-1-mAID
g
MORC-HA Rep1
MORC-HA Rep2
AP2XI-2 -mAID
MORC-2Ty -IAA
MORC-2Ty +IAA
AP2XI-2-mAID-6HA -IAA
AP2XII-1-mAID-6HA -IAA
AP2XI-2-mAID-6HA +IAA
AP2XII-1-mAID-6HA +IAA
Gene model
[0-200]
[0-30]
GRA81
TGME49_243950
AP2IX-9
AP2IX-1

Supplementary Fig. 4 Characterization of the AP2XI-2 containing complexes.

a. Three AP2XI-2 interacting proteins were localized to the nucleus in tachyzoites (32 h post-infection) and bradyzoites (3 days post-infection) and were efficiently depleted when the parasites are treated with IAA. Magenta, anti-IMC1; green, FITC-DBL; red, anti-HA. Scale bar, 10 μm. **b.** Depletion of AP2XII-1 activated the merozoite-specific proteins GRA11A, GRA11B, GRA81, SRS48Q and MIC17A as determined by IFA in the indicated parasites treated with IAA for 3 days, whereas depletion of TGME49_209500, TGME49_214140 and TGME49_275680 did not significantly activate GRA81 expression. Magenta, anti-IMC1; red, anti-Ty. Scale bar, 10 μm. **c.** Volcano plot showing the differentially expressed genes in the AP2XII-1-expressing and AP2XII-1-depleted strain under neutral culture conditions. The merozoite, bradyzoite, sporozoite and tachyzoite highly expressed genes were analyzed from Supplementary data 1. Data from four biological replicates were plotted and a fold change of ≥ 2.0 or ≤ -2.0 with a p-value < 0.05 was deemed statistically significant. **d.** Volcano plot showing the differentially expressed genes in the AP2XII-1-expressing and AP2XII-1-depleted strain under alkaline culture conditions. The merozoite, bradyzoite, sporozoite and tachyzoite highly expressed genes were analyzed from Supplementary data 1. Data from four biological replicates were plotted and a fold change of ≥ 2.0 or ≤ -2.0 with a p-value < 0.05 was deemed statistically significant. **e.** Heat map showing increased expression of merozoite highly expressed genes after depletion of AP2XII-1. The color scale denotes $\log_2$-transformed fold changes. **f.** Heat map showing the decreased expression of tachyzoites highly expressed genes after depletion of AP2XII-1. The color scale denotes $\log_2$-transformed fold changes. **g.** Integrated genome browser screenshots of genomic regions with representative merozoite GRA81 gene and AP2 factors. The data of MORC-HA Rep1 and MORC-HA Rep2 were obtained from the GEO under accession number GSE136060[14].

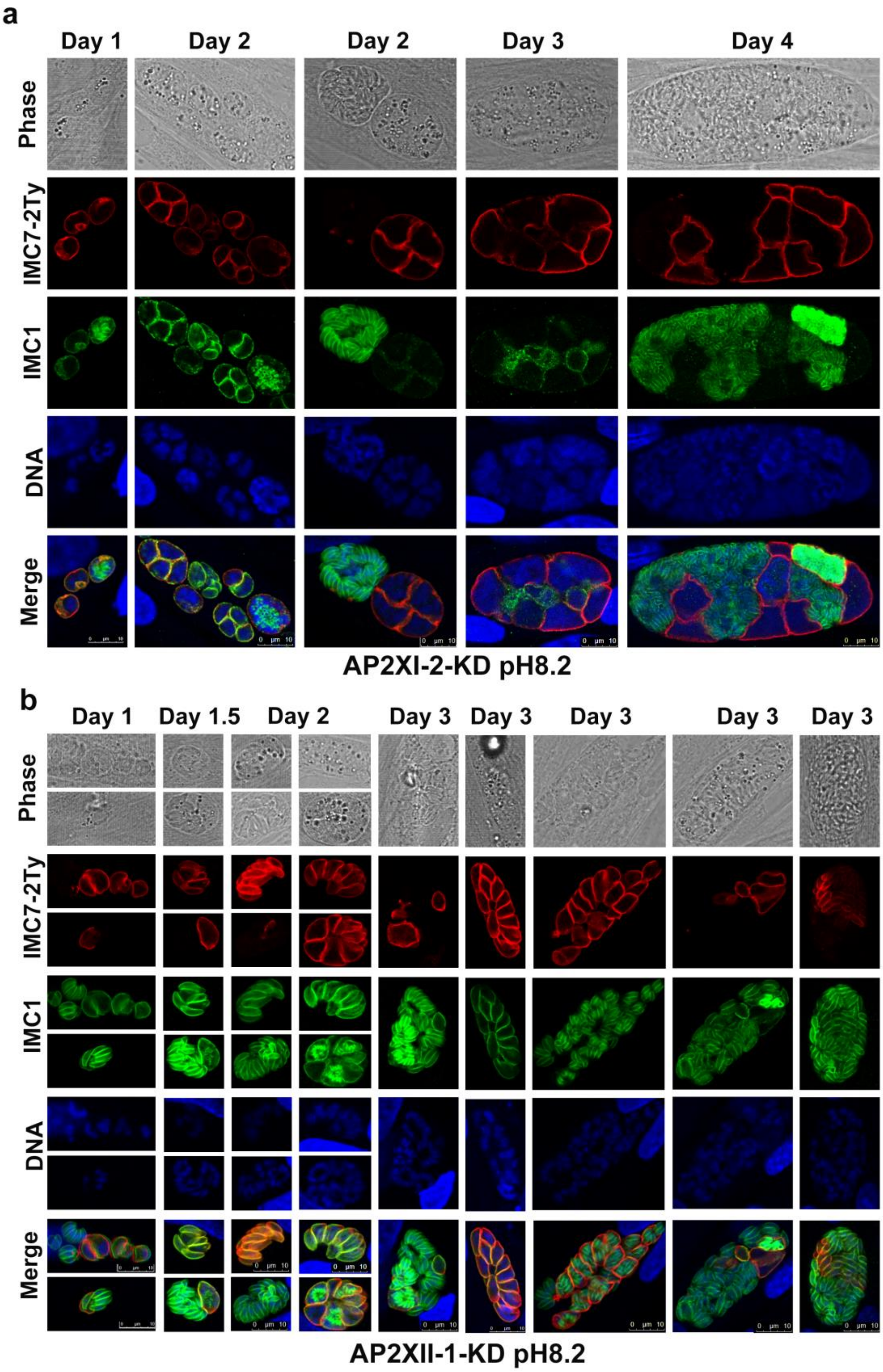

Supplementary Fig. 5 IMC1 and IMC7 proteins dynamics during merogony. Representative images of AP2XI-2 (**a**) or AP2XII-1 (**b**) knock-down (KD) parasites grown in an alkaline medium without CO_2 over 4 days for induction of merozoites. Red, anti-Ty; green, anti-IMC1. Scale bar, 10 µm.

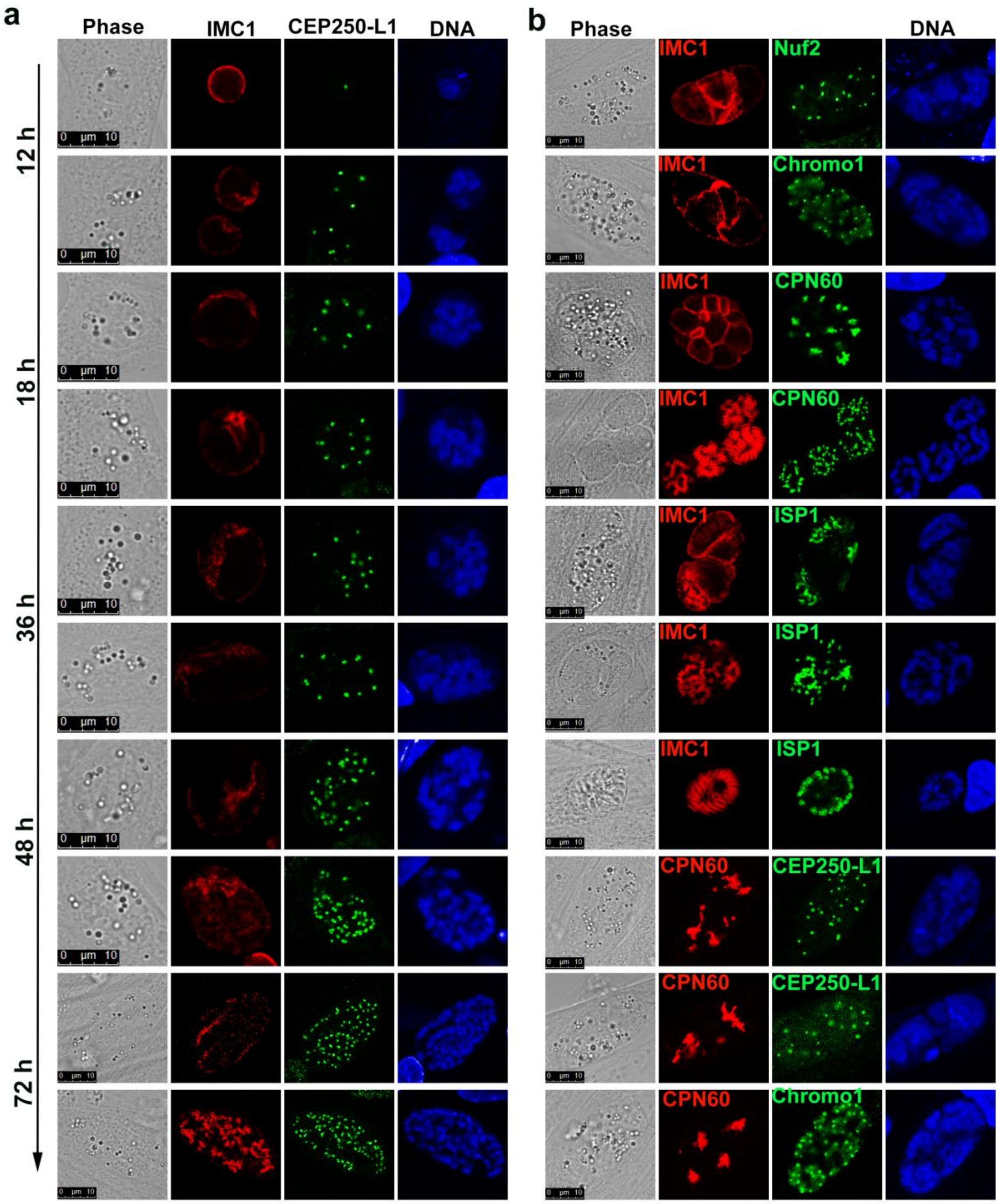

Supplementary Fig. 6 Dynamic analysis of the division of the nucleus of the AP2XI-2-depleted parasites under alkaline culture conditions.

The markers used to analyze nuclear division in the AP2XI-2-depleted included the inner centrosome core marker CEP250-L1-2Ty (**a**), the kinetochore marker Nuf2-2Ty (**b**), the centromere marker Chromo1-2Ty (**b**), the apicoplast marker CPN60 (**b**), and the early apical cap marker ISP1 (**b**). Scale bar, 10 µm.

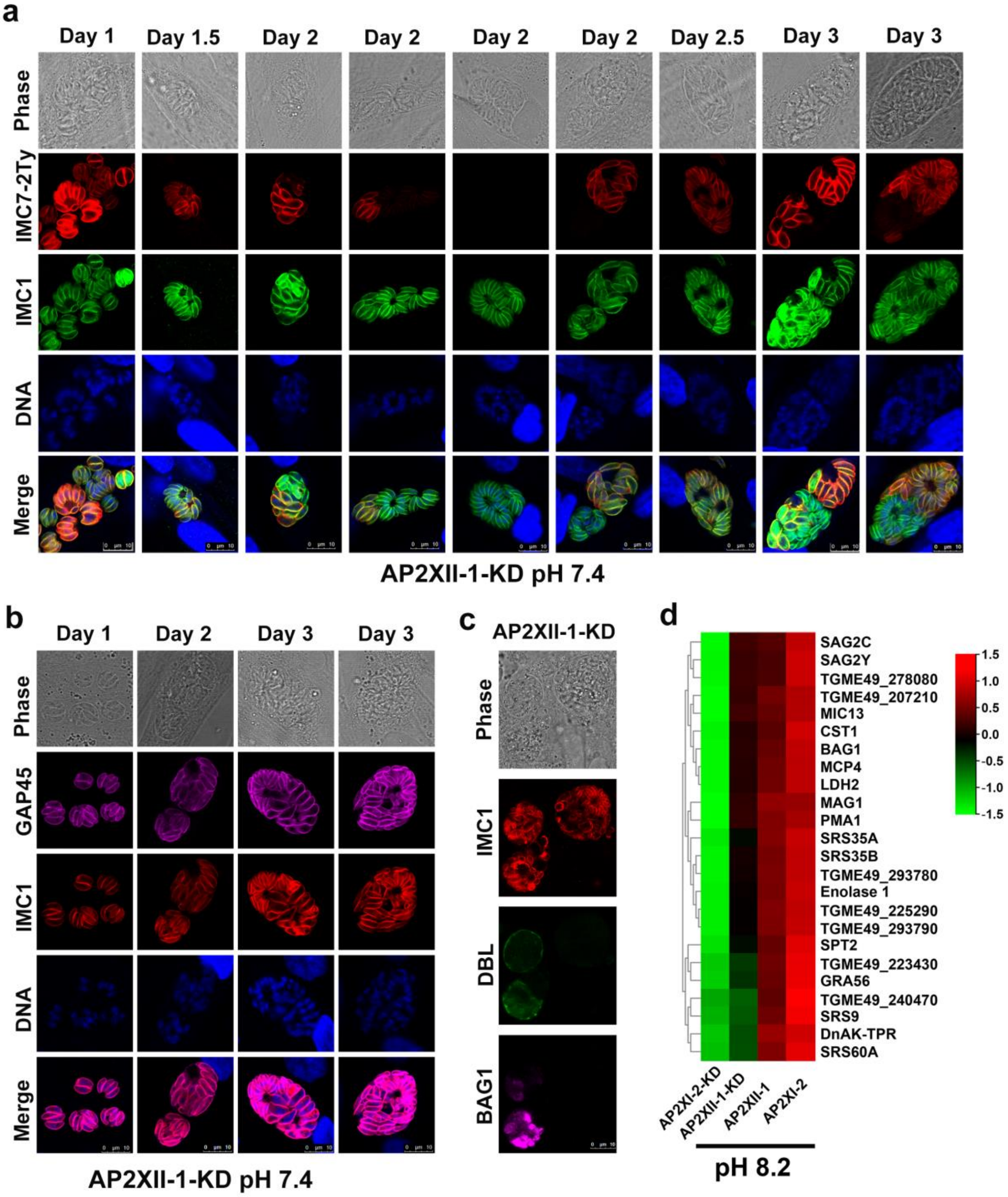

Supplementary Fig. 7 Dynamic analysis of the AP2XII-1-depleted parasites under neutral culture conditions.

a. Representative images of AP2XII-1 knock-down (KD) parasites grown in neutral medium over 3 days and stained with Ty for IMC7 (red) and IMC1 (green). Scale bar, 10 μm. **b.** Representative images of AP2XII-1-KD parasites grown in neutral culture medium over 3 days and stained with GAP45 (magenta) and IMC1 (red). Scale bar, 10 μm. **c.** Representative images of AP2XII-1-KD parasites grown in an alkaline medium without CO_2 for 3 days for induction of bradyzoites. red, anti-IMC1; green, FITC-DBL; magenta, anti-BAG1. Scale bar, 10 μm. **d.** Heat map showing the different expression of selected bradyzoites highly expressed genes after depletion of AP2XI-2 or AP2XII-1. The color scale denotes log_2-transformed fold changes.

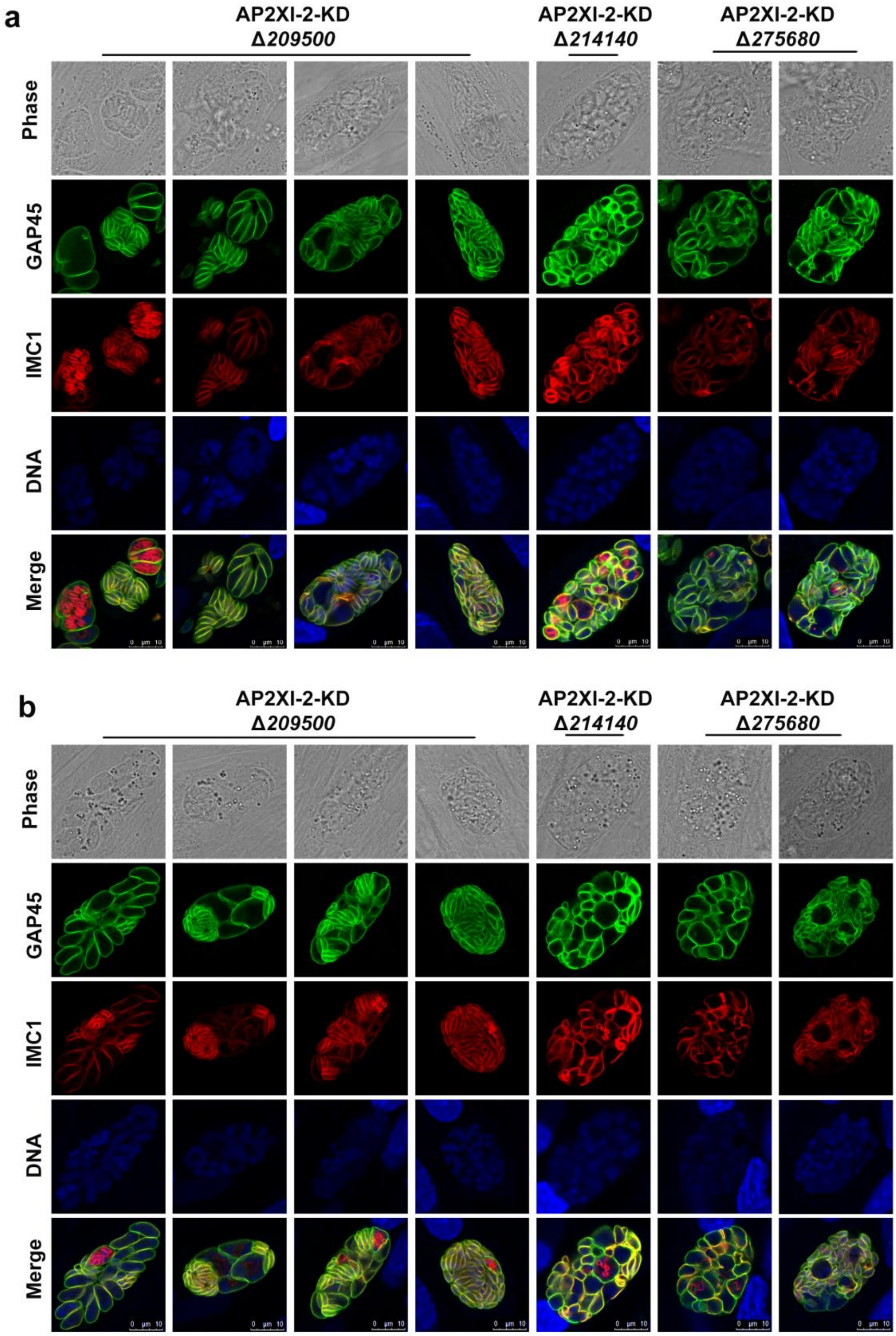

Supplementary Fig. 8 Characterization of the roles of AP2XI-2 interacting proteins (TGME49_209500, TGME49_214140 and TGME49_275680) in the AP2XI-2-depleted parasites.

Representative images of the indicated parasite strains grown in neutral (**a**) or alkaline culture conditions (**b**) for 3 days for induction of merozoites. Green, anti-GAP45; red, anti-IMC1; Scale bar, 10 µm.

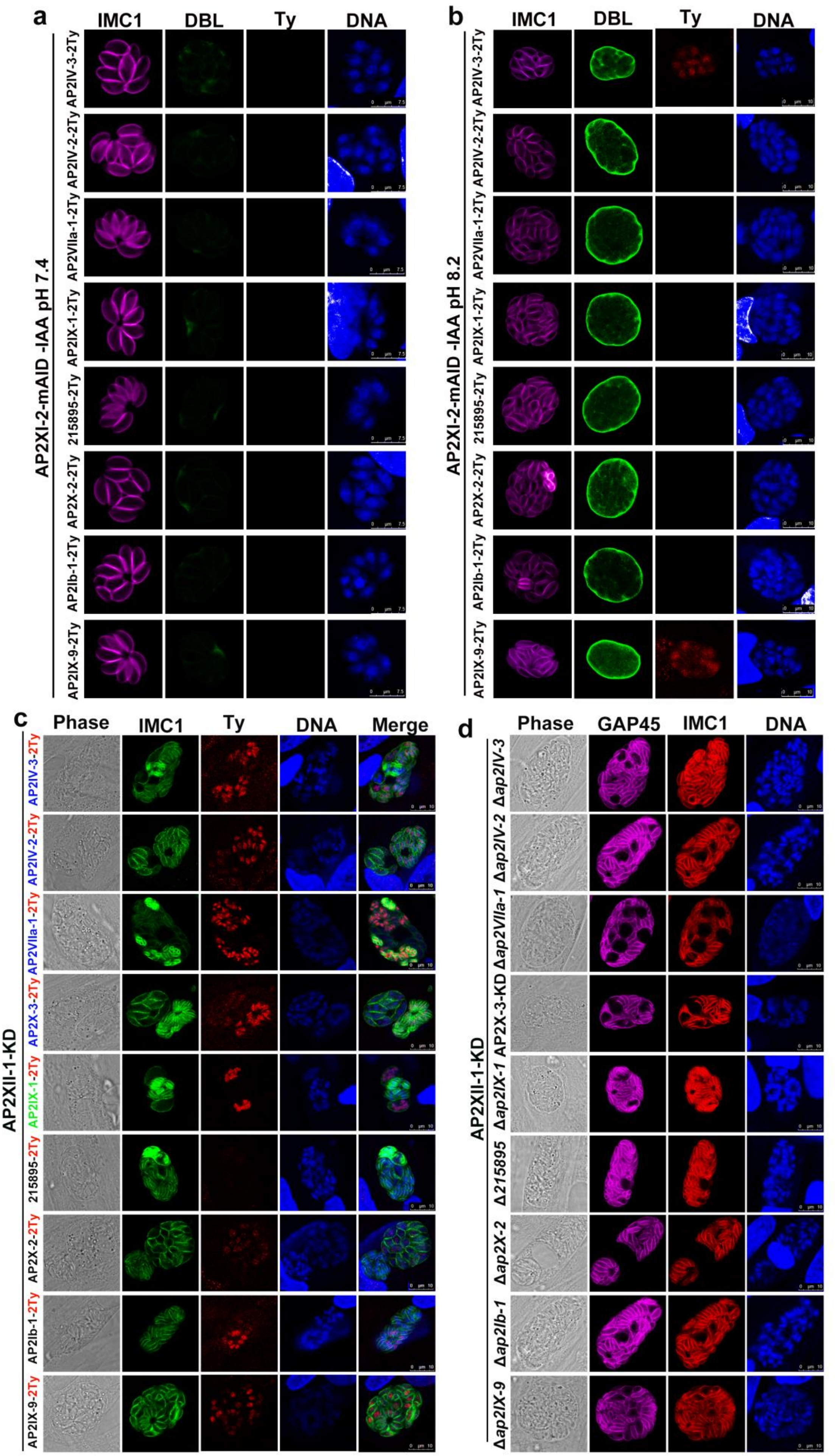

a
IMC1
DBL
Ty
DNA
AP2XI-2-mAID -IAA pH 7.4
AP2IV-3-2Ty
AP2IV-2-2Ty
AP2VIIa-1-2Ty
AP2IX-1-2Ty
215895-2Ty
AP2X-2-2Ty
AP2Ib-1-2Ty
AP2IX-9-2Ty
b
IMC1
DBL
Ty
DNA
AP2XI-2-mAID -IAA pH 8.2
AP2IV-3-2Ty
AP2IV-2-2Ty
AP2VIIa-1-2Ty
AP2IX-1-2Ty
215895-2Ty
AP2X-2-2Ty
AP2Ib-1-2Ty
AP2IX-9-2Ty
c
Phase
IMC1
Ty
DNA
Merge
AP2XII-1-KD
AP2IV-3-2Ty
AP2IV-2-2Ty
AP2VIIa-1-2Ty
AP2X-3-2Ty
AP2IX-1-2Ty
215895-2Ty
AP2X-2-2Ty
AP2Ib-1-2Ty
AP2IX-9-2Ty
d
Phase
GAP45
IMC1
DNA
AP2XII-1-KD
Δap2IV-3
Δap2IV-2
Δap2VIIa-1
AP2X-3-KD
Δap2IX-1
Δ215895
Δap2X-2
Δap2Ib-1
Δap2IX-9

Supplementary Fig. 9 Characterization of the secondary transcriptional regulators in the AP2XI-2-expressing or AP2XII-1-depleted parasite.

a. The expression pattern of the secondary transcriptional regulators in the AP2XI-2-expressing tachyzoites cultured under neutral conditions for 30 h. Magenta, anti-IMC1; green, FITC-DBL, red, anti-Ty. Scale bar, 7.5 µm. **b.** The expression pattern of the secondary transcriptional regulators in the AP2XI-2-expressing bradyzoites induced by exposure to alkaline conditions for 3 days. Magenta, anti-IMC1; Green, FITC-DBL, red, anti-Ty. Scale bar, 10 µm. **c.** Representative images of the confocal immunofluorescence staining showing the expression of the secondary transcriptional regulators during merogony after depletion of AP2XII-1 under alkaline conditions for 3 days. The secondary transcriptional AP2 factors shown in blue and green colors on the left side of the figure denote those mainly expressed in mature merozoites and schizonts, respectively. Factors shown in black color denote those exhibiting slight increase upon depletion of AP2XII-1. Green, anti-IMC1; red, anti-Ty. Scale bar, 10 µm. **d.** Representative image of the indicated parasites grown in alkaline medium for 3 days for induction of merozoites. Magenta, anti-GAP45; red, anti-IMC1. Scale bar, 10 µm.
