# Supplementary material for: The transcription factor AP2XI-2 is a key negative regulator of Toxoplasma gondii merogony
Source: Nat Commun. 2024 Jan 26;15:793. doi: 10.1038/s41467-024-44967-z (PMC10817966; doi:10.1038/s41467-024-44967-z)
Supplement: Supplementary file 3 — Description of Additional Supplementary Files [file 41467_2024_44967_MOESM3_ESM.pdf]

Description of additional supplementary files

Title: Supplementary data 1

Description: Summary of the transcriptomic data analysis of the Pru::AP2XI-2-mAID-6HA and Pru::AP2XII-1-mAID-6HA strains under neutral culture conditions (pH 7.4) or alkaline medium (pH 8.2), with or without IAA.

Title: Supplementary data 2

Description: Summary of the mass spectrometry data analysis of Toxoplasma gondii proteins immunoprecipitated with AP2XI-2-mAID-6HA.

Title: Supplementary data 3

Description: Summary of peak calls from CUT&Tag for AP2XI-2-mAID-6HA, AP2XII-1-mAID-6HA and AP2XI-2-mAID-MORC-2Ty, with or without IAA.

Title: Supplementary data 4

Description: Summary of the transcriptomic data analysis of the selected AP2 factors in the AP2XI-2 and AP2XII-1 depleted parasites.

Titel: Supplementary data 5

Description: Primers and plasmids used in the study
